# The Modern Remedies for Pertussis

**Published:** 1881-12

**Authors:** 


### THE MODERN REMEDIES FOR PERTUSSIS.

Prof. Otto Heubner, of Leipzig, has prepared a statistical table of the effects of some of the modern remedies for whooping-cough, basing it upon as nearly accurate observations as possible. He also places his parallel studies on a level with the methods prescribed by Liebermeister, where, though not yet complete. it is possible, with a smaller number of cases, to compare the two methods of treatment. In one column of the table the author gives an analysis of the effects of the bromide of potassium, quinine, chloral hydrate, salicylic acid and belladonna. The comparative value of potassium bromide is shown by a comparison of twenty-three uncomplicated cases. In not one of these was the duration of the disease shortened by the remedy, but, on the other hand, in a limited number of cases (nine times in twenty-three cases), the intensity of the paroxysms as well as their frequency was diminished.

Of quinine (administered internally in small doses, not exceeding 0.3 per day) Heubner observed that three times in eleven uncomplicated cases, an abreviation of the duration of the disease, while the paroxysms also in five cases underwent a favorable resolution.

Chloral hydrate was given internally in two cases, in divided doses; in all the rest by one enema daily, containing a large quantity (0.3—0.1). The result of this remedy stands in close relation to that of quinine, in so far as a diminution in duration of the disease was observed only twice in ten cases, while a desirable resolution of the individual paroxysms took place six times, and even to a greater degree than under treatment by quinia.

The salicylic acid was given internally but once, being administered at other times by inhalation of a one-half to one-third per cent. solution three times daily, in the dose of thirty grams, by means of

Siegle's apparatus. In the seventeen cases thus treated the effect upon the length of the disease was very unimportant, since only twice could a positive shortening be credited to it. The alleviation of the paroxysms, on the other hand, yielded to this treatment very gratifying results ; for, ten times in the seventeen cases, the diminution of the severity or of the frequency of the seizures was very striking. It was in fact at one time the intensity, at another the frequency which was so favorably reduced, without any perceptible change in the other factors of the disease.

Belladonna was for the most part administered in the form of the powdered extract, and in a few cases in the form of the powdered leaves, the daily dose being 0.15—0.3, and in eight uncomplicated cases, besides the immediate checking of the paroxysms, the duration of the disease itself was cut short by the remedy, the attacks becoming also milder.

Without entering into a discussion of the author's detailed statements and his very plausible calculations, which moreover are very interesting, his statements resolve themselves thus: For the alleviation of the paroxysms, the inhalation of salicylic acid and the chloral hydrate promise the most positive results. For the abbreviation of the entire disease, the greatest benefit is to be expected from belladonna and quinine. Still these results are to be considered only relative.—*Wiener Medizin. Wochenschrift.*—*Cincinnati Lancet and Clinic.*

